# Cigarette Smoke-Induced Reactive Oxygen Species Formation: A Concise Review

**DOI:** 10.3390/antiox12091732

**Published:** 2023-09-07

**Authors:** Yoon-Seok Seo, Jung-Min Park, Jae-Hyeong Kim, Moo-Yeol Lee

**Affiliations:** BK21 FOUR Team and Integrated Research Institute for Drug Development, College of Pharmacy, Dongguk University, Goyang-si 10326, Gyeonggi-do, Republic of Korea; gugod11@dongguk.edu (Y.-S.S.); jmpark@dongguk.edu (J.-M.P.); woguzdhkdwk@dongguk.edu (J.-H.K.)

**Keywords:** smoking, cigarette smoke, reactive oxygen species, oxidative stress, NADPH oxidase

## Abstract

Smoking is recognized as a significant risk factor for numerous disorders, including cardiovascular diseases, respiratory conditions, and various forms of cancer. While the exact pathogenic mechanisms continue to be explored, the induction of oxidative stress via the production of excess reactive oxygen species (ROS) is widely accepted as a primary molecular event that predisposes individuals to these smoking-related ailments. This review focused on how cigarette smoke (CS) promotes ROS formation rather than the pathophysiological repercussions of ROS and oxidative stress. A comprehensive analysis of existing studies revealed the following key ways through which CS imposes ROS burden on biological systems: (1) ROS, as well as radicals, are intrinsically present in CS, (2) CS constituents generate ROS through chemical reactions with biomolecules, (3) CS stimulates cellular ROS sources to enhance production, and (4) CS disrupts the antioxidant system, aggravating the ROS generation and its functions. While the evidence supporting these mechanisms is chiefly based on in vitro and animal studies, the direct clinical relevance remains to be fully elucidated. Nevertheless, this understanding is fundamental for deciphering molecular events leading to oxidative stress and for developing intervention strategies to counter CS-induced oxidative stress.

## 1. Introduction

Smoking is widely recognized as a significant risk factor for numerous diseases, including cardiovascular conditions, respiratory illnesses, and various types of cancers [1]. The toxicological implications of smoking are attributed to a number of mechanisms, many of which involve intricate molecular events. Thus, despite our increasing understanding, the molecular mechanisms continue to be a focus of rigorous investigation. Oxidative stress is widely identified as one of the key molecular events mediating the pathogenesis of smoking-associated diseases [2,3].

Oxidative stress represents a physiological condition characterized by an imbalance between oxidative and antioxidative potentials [4]. Such imbalance typically involves an excessive or deregulated production of prooxidants and/or a dysfunction within the antioxidant system. Prooxidants, which incite oxidative stress, comprise reactive oxygen species (ROS), radicals, and other oxidizing agents. In contrast, the antioxidant system involves both enzymatic antioxidants like superoxide dismutase (SOD), catalase, and glutathione (GSH) peroxidase, and non-enzymatic antioxidants such as GSH, carotenoids, and vitamins C and E [5].

Prooxidants, typified by ROS and radicals, represent highly reactive molecules that function as oxidizing agents in redox reactions. ROS, the primary endogenous prooxidants, are derivatives of molecular oxygen (O_2_) exhibiting greater reactivity compared to O_2_ itself [6]. These include species such as superoxide (O_2_^•−^), hydrogen peroxide (H_2_O_2_), hypochlorous acid (HOCl), and peroxynitrite/peroxynitrous acid (ONOO^−^/ONOOH). These are produced endogenously through sources like the mitochondrial electron transport chain (ETC) and enzymes including NADPH oxidase (NOX), xanthine oxidase, nitric oxide synthase (NOS), and cytochromes P450 [7,8,9,10,11]. Moreover, they are generated via chemical reactions that involve transition metals like Fe and Cu [12]. Exogenous factors, including air pollutants and radiation, can also induce or stimulate their production [12]. The genesis of oxidative stress is not solely due to excessive or unregulated ROS formation but also linked to the dysregulation of the antioxidant system. Decreased antioxidant capacity can exacerbate ROS production and make biomolecules more susceptible to damage from prooxidants [13]. It is pertinent to mention that ROS, traditionally deemed hazardous byproducts of physiological processes or xenobiotics, also serve as signaling molecules, regulating various redox signaling involved in physiological processes [14]. ROS play crucial roles in diverse cellular events, such as growth, proliferation, differentiation, and apoptosis, by modulating redox-sensitive signaling molecules [14]. Furthermore, excessive ROS can inflict direct and nonspecific damage to various biomolecules, including lipids, proteins, and nucleic acids [13].

A plethora of tobacco products exists, among which cigarettes hold the preponderance, representing over 80% of total global tobacco product revenue [15]. Cigarette smoke (CS) generation has been standardized by regulatory bodies such as International Organization for Standardization or national governments, and there are consensuses in the research community on the trapping methods for CS, at least to some extent [16]. As a consequence, cigarettes are the most extensively researched form of tobacco products. Hence, the literature reviewed in this paper primarily focused on studies exploring CS and its related preparations.

CS is a generic product of cigarette combustion, encompassing an array of over 7000 chemicals [17]. Cigarettes themselves comprise a combination of various additives, such as flavorings, nicotine controllers, and combustion aids, in addition to components like cigarette paper, side seam adhesive, and printing ink, along with a blend of tobacco leaves [18]. This suggests that the composition of CS varies significantly depending on the specific cigarette product and can also be influenced by factors such as combustion temperature. Moreover, an array of CS preparation methods exist, thereby contributing to further variations in its composition [19]. Consequently, it is standard practice for studies to specify the particular cigarette products employed and describe the associated CS preparation methods. Nevertheless, a comprehensive understanding of the compositional differences among cigarette products and their preparations remains incomplete. Therefore, this review will not differentiate between various types of cigarette products and CS preparations as referenced in the studies.

**Figure 1:**
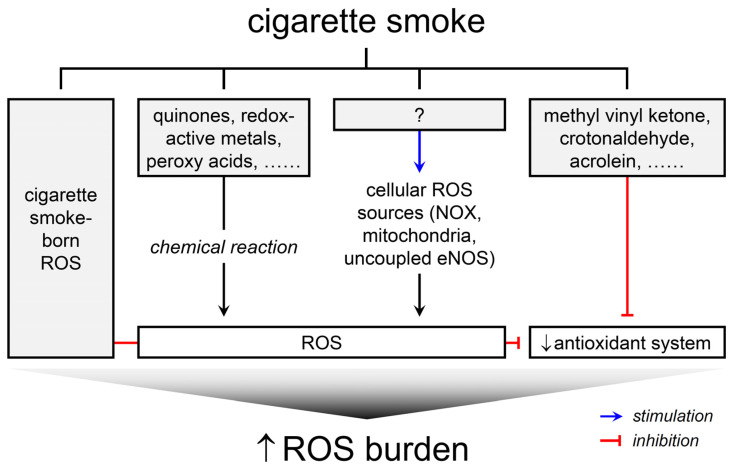
Schematic representation of four principal ways through which CS imposes ROS burden on biological systems.

The primary aim of this review was to investigate the induction of ROS in biological systems by CS, without delving into the biological or toxicological implications of ROS generation or oxidative stress. Numerous studies have probed into the phenomena of CS-induced ROS formation. Collectively, they suggest four principal ways through which CS imposes ROS burden on biological systems: (1) ROS, as well as radicals, are intrinsically present in CS, (2) CS constituents can induce ROS generation via chemical reactions with biomolecules, (3) CS can stimulate endogenous ROS sources in cells to produce ROS, and (4) CS can interfere with antioxidant systems, thereby exacerbating the production or functions of ROS (Figure 1). It is pertinent to note that cellular ROS sources responsive to CS include NOX, mitochondria, and NOS. While the amplification of ROS formation may be a secondary consequence of chronic pathological conditions like inflammation and hyperglycemia induced by CS, as evidenced in animal and human studies [20,21], this review refrains from delving into such complexities. The bulk of the data discussed herein originate from studies utilizing cell or cell-free systems and animal models, leaving the clinical relevance of these ROS formation pathways somewhat nebulous. Nonetheless, understanding these mechanisms is pivotal in deciphering the molecular events leading to oxidative stress and in formulating strategies to alleviate the oxidative stress associated with smoking.

## 2. ROS Formation or Oxidative Stress in Smokers

Elevated oxidative stress, manifested by an intensified production of ROS, is a hallmark characteristic of smokers [4,8,22,23]. Such an observation is primarily grounded in clinical studies showcasing altered profiles of oxidative stress biomarkers in individuals who smoke. The majority of these biomarkers originate from the oxidative modification of biomolecules, encompassing lipid peroxidation products like 4-hydroxynonenal (4-HNE) [24], malondialdehyde (MDA) [25], and 8-isoprostane [26]; protein byproducts altered through oxidation such as protein carbonyls, 3-nitrotyrosine [27], and oxidized α-1 antitrypsin [28,29]; oxidized nucleic acid metabolites, such as 8-hydroxy-2′-deoxyguanosine (8-OHdG) [30]; and the antioxidant levels, notably GSH [31,32]. These biomarkers have been identified and quantified in an array of biological specimens including urine, blood, epithelial lining fluid, sputum, and saliva.

These oxidative stress biomarkers exhibit a tendency toward elevated levels within the respiratory system, the primary site of exposure to cigarette smoke [33,34,35]. For instance, 8-OHdG levels in the lung were discovered to be significantly higher in smokers than nonsmokers, with the degree of this elevation proportional to the cigarette smoking volume [33]. MDA levels were found to be increased in lung tissue samples from patients diagnosed with lung cancer [36]. Additionally, amplified levels of 8-isoprostane and 3-nitrotyrosine were observed in saliva and bronchial mucosa, respectively, from both asymptomatic smokers and patients diagnosed with chronic obstructive pulmonary disease (COPD), a common smoking-related condition [37,38]. 

Apart from the primary exposure site, biomarkers in blood and urine have also been examined to evaluate systemic oxidative stress [39]. Notably, the levels of MDA [39], 8-isoprostane [40,41], 3-nitrotyrosine [42], carbonyl content [43], and oxidized α-1 antitrypsin [28,29] in blood plasma or serum were higher in smokers compared to nonsmokers. Correspondingly, elevated urinary levels of 8-isoprostane [42], MDA [44], and 8-OHdG [45,46] were reported in smokers, demonstrating a quantitative correlation between the number of cigarettes consumed daily and the increment of these biomarkers [46]. It is crucial to note that the biomarker levels in blood or urine are influenced by both excretion and formation. Chronic smoking is known to impair renal function, which could potentially elevate these biomarker levels due to reduced excretion, in addition to the increased formation [30]. Therefore, kidney function must be factored into the interpretation of these findings. For example, the 8-isoprostane/creatinine ratio presents an effective index for oxidative stress assessment [42]. Moreover, high levels of 4-HNE and 8-OHdG have been identified in placenta samples from smoking mothers [47], indicating an association between maternal smoking and intrauterine oxidative stress. Smoking induces oxidative stress not only locally within the respiratory system but also at a systemic level.

Studies have substantiated the detrimental impact of smoking on the antioxidant system, precipitating a reduction in Trolox equivalent antioxidant capacity in plasma, an index of antioxidant capacity [48,49]. The literature abounds with evidence illustrating the adverse effects of smoking on antioxidants including, but not limited to, vitamins A, C, and E, carotene, and soluble thiol pools such as GSH [50,51]. A majority of these investigations have reported a decrease in these antioxidants consequent to smoking. However, a subset of studies has documented negligible alterations or even augmentation in GSH levels, potentially attributable to a compensatory mechanism countering depletion or a rebound phenomenon [52,53]. Although complicate molecular mechanisms are implicated, Nrf2 appears to play crucial roles in this compensation by upregulating enzymes responsible for GSH biosynthesis and regeneration [54]. Additionally, smoking has been found to suppress or downregulate enzymatic antioxidant systems including SOD and catalase in erythrocytes and plasma [55,56], as well as extracellular SOD in serum [57].

Given that the bulk of these studies have focused on chronic smokers, the perturbations in these biomarkers may represent the fallout of extended exposure to CS, suggesting that CS-induced oxidative stress could be a sequel to prolonged incidents such as smoking-induced inflammation or metabolic disorders. Nonetheless, even transient exposure to CS has been sufficient to amplify biomarkers of oxidative stress. As an example, the consumption of a single cigarette led to an upsurge in 8-isoprostane and soluble NOX2-derived peptide, a marker for NOX2 activation, along with a decrease in NO bioavailability and vitamin E in serum within 30 min [58,59]. Correspondingly, 8-OHdG levels in peripheral leukocytes were noted to rise a mere 10 min after smoking two cigarettes [60]. Passive smoking too has been found to increase plasma 8-isoprostane levels in nonsmokers within 30 min [61]. The rapid escalation in these biomarkers suggests an immediate induction of ROS formation by CS, irrespective of the presence of pathological conditions [62]. This notion is bolstered by findings that demonstrate the restoration of biomarker levels following smoking cessation, even in the absence of symptom improvement in patients with diabetes, hypercholesterolemia, or hypertension [63].

Regrettably, few studies have successfully detected a direct increase in ROS in reasonable ways rather than simply observing oxidative stress markers in human subjects, presumably due to the inherent technical challenges associated with measuring ROS. A pioneering study detected chemiluminescence in the blood plasma of smokers, which promptly ceased following smoking cessation, leading researchers to postulate its origin in ROS such as singlet oxygen derived from CS [64]. Another investigation, which analyzed expired breath condensate, reported elevated levels of hydrogen peroxide in smokers compared to nonsmokers, indicative of CS-induced hydrogen peroxide formation in the airway epithelial lining fluid [65]. This collective body of evidence intimates a direct elevation of ROS as a result of smoking.

## 3. The Ways How CS Imposes ROS Burden on the Biological Systems

The current understanding of how CS induces or stimulates ROS formation principally draws upon data procured from cells and cell-free systems rather than clinical or in vivo studies [6]. The study of CS inherently demands CS or its preparations. Emissions from a lit cigarette divide into two fractions, mainstream and sidestream smokes. However, the term CS typically refers to mainstream smoke. CS is characterized as an aerosol comprising minuscule particulates suspended in a blend of gases [17,66]. The combustion of cigarettes yields CS, which can subsequently be analyzed directly or exposed to subjects. Alternatively, CS can be captured by entrapping it on a Cambridge filter pad or by bubbling it in liquid mediums such as aqueous buffers or organic solvents. The former is referred to as total particulate matter (TPM) in scientific literature [67], which embodies the particulate phase of CS, inclusive of particles larger than 0.1 µm but typically excluding extremely volatile compounds. TPM is often retrieved from a filter pad using organic solvents for experiments. Tar customarily refers to the nicotine-free, dry TPM [68]. On the other hand, the fractions of CS that pass through the Cambridge filter pad is referred to as gas vapor phase. It entails particles smaller than 0.1 µm suspended within a mix of gases. The cigarette smoke extract (CSE) is prepared by bubbling gas vapor phase or whole CS in liquid mediums. Both CSE and TPM are commonly utilized as CS derivatives in experimental settings. Various CS forms or preparations have been employed across different studies. Nevertheless, not so much information has been gained from CS-related ROS studies in biological systems by differentiating between them, while difference in their chemical composition has been substantially characterized. Consequently, specific details regarding the type of CS were not distinguished in the following discussion.

### 3.1. Radicals and ROS Present in CS

CS is a complex mixture encompassing over 7000 chemicals [17,18]. Among these, it contains substances of high reactivity that possess the ability to instigate oxidative stress, including radicals and putative ROS [1,69]. CS has been suggested to contain unstable radicals [70], and consequently, a range of radicals has been identified therein. These include carbon-centered radicals such as alkyl radicals, oxygen-centered radicals such as peroxy and alkoxy radicals, and nitrogen-centered radicals such as nitric oxide [71]. The specific species of radicals detected in CS can vary depending on the chosen analytical methods and CS preparations.

Initial investigations using electron spin resonance spectroscopy (ESR) in conjunction with a spin trap detected carbon-centered and oxygen-centered radicals in the gas phase of CS [72]. Oxygen-centered radicals were hypothesized to be phenoxy, alkoxy, aryloxy, and alkylperoxy radicals. Subsequent studies identified alkoxy radicals (·OR) such as ·OCH_3_, ·OC_2_H_5_, ·OC_3_H_7_, and ·OC(CH_3_)_2_C_6_H_5_, in the gas phase [73]. Carbon-centered radicals were identified in CS by deploying a highly selective, kinetically rapid spin trap in conjunction with ESR, high performance liquid chromatography (HPLC)/nuclear magnetic resonance (NMR), and mass spectroscopy. These include carbonyl radicals (·CO-R) such as ·CO-CH_3_, ·CO-C_2_H_5_, ·CO-C_3_H_7_, ·CO-C_4_H_9_, and alkyl amino carbonyl radicals (·CO-NH-R) such as ·CO-NH-CH_3_, ·CO-NH-C_2_H_5_, ·CO-NH-C_3_H_7_, ·CO-NH-C_4_H_9_, ·CO-NH-C_5_H_11_ [74,75]. Alkyl amino carbonyl radicals are believed to be secondary radicals, generated from other reactive radicals and nitric oxide in CS. These radicals could be detected in aged CS as they are less reactive and can persist for several minutes [74,75]. The studies identified relatively short-lived radicals as the analysis was conducted with the gas phase immediately prepared from a flow-through. Nonetheless, radicals with extremely short half-life were not likely to be detected in these analyses.

The long-lived radicals *o*- and *p*-benzosemiquinones were identified in aqueous extracts of cigarette tar (ACT) prepared from cigarette products [76,77,78]. These long-lived radicals were regarded as secondary products, produced from chemical reactions between short-lived radicals and organic substances [79].

The combustion of organic compounds yields highly reactive, unstable radicals such as superoxide, hydroxyl radical, and non-radical ROS like singlet oxygen [71,72]. Given their extremely short half-lives, typically less than a millisecond, they are unlikely to be detected in CS or CS preparations [80]. Nonetheless, these short-lived radical ROS, as well as hydrogen peroxide, could be detected in ACT [77,81,82,83]. These ROS seem to derive from chemical reactions between long-lived radicals and O_2_ in ACT, rather than being native to CS, as their detection in ACT under anaerobic conditions is minimal [77,83]. While theoretically generated by cigarette combustion [81,84], the detection of superoxide and hydroxyl radical in CS poses significant challenges. It is a common literary assertion that CS contains ROS, though the interpretation of this claim may vary from its literal interpretation.

While radicals and ROS are present in CS, radicals with extremely short half-lives may not reach the respiratory system [80]. The number of radicals and ROS that may penetrate into the circulation is likely constrained due to their stability and potential reactivity within the first line of defense barrier. Further investigations are warranted to elucidate the bioavailability and toxicological implications of the radicals and ROS present in CS.

### 3.2. ROS Formation in Biological Systems via Chemical Reactions Involving CS Constituents

In addition to inherent ROS in CS, CS generates ROS through chemical reactions among its redox-active constituents with biological environments. Redox-active constituents like benzosemiquinones [77], benzy[a]pyrene [85], α,β-unsaturated carbonyls [86], peroxides and peroxy acids [87], and metal ions [88] are important examples. Benzosemiquinones, with extended half-lives, can penetrate the blood–air barrier and gain access to the circulation, thereby systemically producing superoxide through quinone redox cycling and forming adducts with biomolecules, such as hemoglobin and albumin [71,89,90]. Meanwhile, benzo[a]pyrene undergoes microsomal metabolism to form quinones, which also lead to superoxide production via the quinone cycle [85]. α,β-Unsaturated carbonyls such as acrolein, crotonaldehyde, and methyl vinyl ketone, also referred to as reactive carbonyl species, are reactive electrophiles that produce singlet oxygen through their interaction with peroxynitrite [86]. Intriguingly, CS preparations generate superoxide in aqueous media like biological buffer solutions, cell culture media, and blood plasma [87]. While not all constituents were identified, peroxidase substrates such as peroxides and peroxy acids are postulated to react with bicarbonate anion in aqueous environments to yield superoxide.

Metal ions in CS, acting as secondary sources of ROS, deserve mention. CS contains a diverse range of heavy metals and metalloids, originating from tobacco leaves and other components of cigarettes such as printing ink, wrap papers, paper adhesives, and filters, in the form of impurities [88]. Upon combustion, these metals become inhalable aerosols. CS metal species include As, B, Ba, Br, Cd, Cl, Cs, Cu, Fe, Hg, I, K, Li, Mn, Na, Pb, Rb, Sb, Sn, Tl, and Zn [88,91,92], with no significant differences observed between different cigarette products [93]. Most trace metals are contained in the particulate phase, with Hg uniquely detected in the gas phase [91]. Among these, transition metals play a significant role in ROS generation [17,94] due to their ability to exist in various oxidation states, thereby facilitating electron donation or acceptance and catalyzing redox reactions to produce ROS [95]. Specifically, Fe^2^⁺ promotes the formation of hydroxyl and hydroperoxyl radicals from hydrogen peroxide through the Fenton reaction. Similarly, Cu^2^⁺ generates hydroxyl radicals through a Fenton-like reaction with hydrogen peroxide [96] and also participates in chemical reactions yielding benzoquinone, which facilitates superoxide production [95]. Cr exists in several oxidation states, with both Cr^3^⁺ and Cr^6^⁺ capable of producing superoxide [97], and Cr^6^⁺ additionally generating hydroxyl radicals through a Fenton-like reaction [98]. Ni^2^⁺ catalyzes the production of hydroxyl radicals via the Haber-Weiss reaction [99,100]. Cd and Pb are not generally redox-active but contribute to oxidative stress by interfering with the cellular antioxidant system [88]. These metal ions react with cysteine, thereby depleting the antioxidant thiol pool. Furthermore, the concentration of metals like Al, As, Cd, Cr, Cu, Fe, Hg, Pb, Ni, and Zn has been detected at higher levels in blood and tissue samples from smokers compared to nonsmokers [101,102,103,104,105], suggesting that metal ions in CS could be a significant source of ROS in smokers [106].

### 3.3. ROS Production from Cellular Sources Stimulated by CS

#### 3.3.1. NOX

NOX, a membrane-associated enzyme complex, facilitates the transfer of electrons from NADPH to O_2_, generating ROS. Distinct from other ROS-producing enzymes, NOX is dedicated exclusively to ROS production, distinguishing it as a unique component in cellular metabolism [107]. First identified in phagocytic leukocytes such as neutrophils and macrophages, NOX contributes to the respiratory burst during immune responses. The enzyme complex comprises membrane-associated catalytic subunits, gp91phox and p22phox, alongside regulatory cytosolic subunits including p47phox, p67phox, p40phox, and Rac. In the non-phagocytic cells, NOX organizing protein 1 (NOXO1) and NOX activating protein 1 (NOXA1) can replace p47phox and p67phox, respectively. Under normal conditions, NOX remains dormant; however, upon activation, the regulatory cytosolic subunits translocate to the membrane, where they interface with the membrane-bound catalytic subunits. The assembled enzyme complex produces superoxide through the single electron reduction in O_2_, using NADPH as the electron donor. NOX activity has been detected in nonphagocytic cells, and further isoforms of gp91phox have been identified. To date, seven human NOX isoforms have been categorized, labeled as NOX1-5, and dual oxidase (DUOX) 1 and 2. The phagocytic NOX is designated as NOX2. Each NOX isoform displays tissue-specific expression patterns, unique subcellular localizations, and interacts with distinct regulatory subunits, hence bearing its own regulatory mechanism and pathophysiological function. Excessive NOX activity can result in redox imbalance, thus culminating in oxidative stress [108].

The pathophysiological functions of NOX have been a research focus for years. More recently, a growing number of studies have begun to illuminate its role in ROS production and oxidative stress induced by chemical and physical stressors, such as heavy metals, organic solvents, ionizing radiation, and ultraviolet radiation [109,110,111]. Therefore, NOX is proposed as a key intermediary in oxidative stress responses triggered by xenobiotic substances.

NOX is activated by CS to produce ROS in a variety of cell types, including lung epithelial cells [112,113], tracheal and arterial smooth muscle cells [114,115,116], vascular endothelial cells [109,117,118,119], cancer cells [120,121], as well as macrophages and neutrophils [122]. Consistent findings have been reported in isolated organ studies, with CS shown to stimulate NOX-dependent ROS production in isolated rat artery [123]. Evidence from animal studies has corroborated increased NOX activity with elevated basal ROS levels in lung tissue and bronchoalveolar lavage fluid (BALF) cells from CS-exposed mice [113,124,125]. These elevations were mitigated by conditional knockdown of NOX1, 2, and 4, and deletion of NOX2. Support for CS-induced, NOX-mediated ROS production largely comes from studies employing NOX inhibitors such as diphenyleneiodonium (DPI), apocynin, and VAS2870 [116,122,126]. However, it is important to note that many of these inhibitors suffer from a lack of specificity for NOX. DPI, for instance, inhibits flavoproteins and, thus, impacts the mitochondrial electron transport chain [127]. The effectiveness of apocynin is limited as it requires myeloperoxidase-mediated metabolic activation to form a dimer [128]. Apocynin possesses hydrogen peroxide scavenging activity as well. Furthermore, VAS2870 exhibits numerous off-target effects [129,130]. Conventional NOX assays measuring ROS also do not permit distinction between the activities of individual NOX isoforms. As such, to conclusively ascertain the role of NOX, genetic approaches are essential, complementing the use of pharmacological inhibitors. 

There exists a relatively limited number of studies that have conclusively identified the isoforms responsible for CS-stimulated ROS generation, possibly due to the lack of an isoform-selective NOX inhibitor. Of the seven identified NOX isoforms, NOX1 and NOX2 emerged as the primary isoforms responsive to CS exposure [114,115,116,117,119,122]. CS-induced superoxide production appears attenuated with the knockdown of NOX1, whereas the knockdown of NOX4 has a minimal impact on ROS generation in vascular smooth muscle cells [116]. The deletion of either gp91phox or p47phox effectively impedes ROS production in tracheal smooth muscle cells [114,115,117], bone marrow-derived macrophage [122], and cardiac endothelial cells [119], thereby highlighting the significant contribution of NOX2.

The mechanisms underpinning NOX activation by CS exposure remain partially elucidated. The tyrosine kinase c-Src has been proposed as a target for CS-triggered NOX activation in several studies [114,117,118]. c-Src activation by CS and the subsequent prevention of NOX activation and ROS production by a c-Src inhibitor have been observed in both brain endothelial cells [118] and tracheal smooth muscle cells [114,117]. The activation of c-Src appears to stimulate NOX2 by promoting the phosphorylation and translocation of p47phox, a subunit integral to the assembly and activation of NOX2, in tracheal smooth muscle cells [117]. Additionally, Toll-like receptor 4 (TLR4) has been proposed as a potential upstream signaling molecule responsible for the CS-induced activation of c-Src in tracheal smooth muscle cells [115]. Thus, the TLR4-MyD88-c-Src signaling axis may serve a central role in mediating NOX2 activation by CS. 

CS has also been found to prompt an intracellular Ca^2+^ elevation, which subsequently activates protein kinase C (PKC). This activation, in turn, results in NOX-dependent ROS generation in glioma cells [120]. PKC appears to activate NOX via the phosphorylation and translocation of p47phox [131,132]. Among isozymes, PKCα, PKCδ, and PKCε have been found to respond to CS and subsequently activate NOX in either glioma or brain endothelial cells [118,121]. Specifically, PKCα has been implicated in Ca^2+^-dependent NOX activation, while the role of Ca^2+^ in the activation of PKCδ and PKCε by CS remains to be clarified [118,121]. 

Although the specific constituents responsible for NOX activation are yet to be fully determined, α,β-unsaturated aldehydes such as acrolein have been suggested as potent candidates for NOX activation in CS. Acrolein has been shown to stimulate DPI-inhibitable superoxide production in arterial endothelial cells [109]. It is suggested that acrolein, and potentially other potent nucleophiles present in CS, may activate NOX by impacting sulfhydryls and disulfide bonds, thereby modifying the conformation and functionality of NOX.

The activation of NOX by CS is notably augmented under pathological conditions. Specifically, the pro-inflammatory cytokine IL-1β has been shown to enhance CS-induced NOX activation via a p47phox-dependent mechanism, whereas the anti-inflammatory lipid mediator PGI_2_ can inhibit p47phox translocation and consequent NOX activation by CS [119]. Concurrently, the activation of kinin B1 receptor, a G protein-coupled receptor that mediates the inflammatory response in COPD caused by smoking, further intensifies CS-induced NOX activation through yet unidentified mechanisms [133]. Notably, CS has been demonstrated to upregulate both IL-1β and the kinin B1 receptor [112,133]. Taken together, IL-1β and kinin B1 receptor act as potentiating factors for CS-induced NOX activation.

It is important to note that NOX isoforms are not ubiquitously expressed; each isoform displays differential expression across organs and cells [107]. For example, while NOX2 predominates in professional phagocytes, NOX3 is largely exclusive to the inner ear, and lung epithelial cells express NOX1, NOX2, NOX4, and NOX5. Moreover, rodent models do not express NOX5 [108]. Thus, the lack of involvement of NOX isoforms other than NOX1 and NOX2 in CS-induced ROS generation could be due to insufficient information, rather than a lack of relevance. Consequently, the influence of CS on other NOX isoforms remains an area of active inquiry.

In addition to enhancing NOX activation, CS has also been observed to upregulate NOX expression levels. Most NOX isoforms, with limited data available for NOX3 and NOX5, exhibited increased expression in cells exposed to CS [113,123,124,134,135,136,137]. In animal models, NOX1, 2, 4, and NOXO1 were found to be induced in the lungs of mice [113,124], NOX2 in rat arteries [123], and NOX4, p22phox, and DUOX1 in rat brains [138]. Similarly, lung tissues from smoking COPD patients demonstrated the upregulation of NOX1, 2, 4, and 5 [124]. While this upregulation of NOX expression is anticipated to correlate with increased activity, further validation is warranted. 

Contrary to the majority of studies reporting NOX activation, a part of investigations documented NOX inhibition by CS [139]. This study found that CS impeded ROS generation that was stimulated by phorbol 12-myristate 13-acetate and the translocation of p67phox in neutrophils isolated from nonsmokers. While the detailed molecular mechanism for this inhibition remains elusive, reactive aldehydes such as acrolein in CS were proposed as potential NOX inhibitors in neutrophils. This suggestion, however, stands in contrast to previous study proposing acrolein as a potential NOX activator [109]. This discrepancy in the impact of CS or acrolein on NOX may arise from variations in the strength of CS exposure [139], underscoring the need for further research. Intriguingly, a study observed an amplified pulmonary inflammatory response in p47phox- or gp91phox-null mice [125], contradicting the prevalent notion that NOX2 typically promotes inflammatory processes. The genetic ablation of p47phox or gp91phox resulted in an augmented inflammatory response to CS in the lung, even in the presence of reduced ROS production in BALF cells. This heightened susceptibility to CS may be attributable to defects in phagocytosed particle degradation and the impaired turnover of inflammatory cells in the lesion [125]. Therefore, it is important to note that the role of NOX under the influence of CS is not universally pro-inflammatory.

#### 3.3.2. Mitochondria

Mitochondria generate cellular ATP via oxidative phosphorylation, also referred to as electron transport-linked phosphorylation. This process entails a series of electron transfers executed by the ETC, resulting in the establishment of a proton gradient across the mitochondrial inner membrane. This gradient functions as the driving force behind ATP synthesis. The ETC transfers electrons from carriers such as NADH and succinate/FADH_2_ to O_2_. However, an untimely electron leak from ETC complexes I, II, or III can initiate a one-electron reduction in O_2_, thereby forming superoxide [140]. Physiologically, approximately 2% of electrons leak from the ETC to the oxygen molecule [141,142,143]. Beyond these complexes, other sites of superoxide production have been identified, with the total number rising to 12 [140]. Such electron leakage tends to increase in environments characterized by high levels of electron carriers or elevated oxygen tension [141]. Consequently, xenobiotics can stimulate superoxide production in mitochondria.

Mitochondria represent dynamic organelles that continuously undergo coordinated cycles of fission and fusion, a process known as “mitochondrial dynamics” [144]. These mechanisms govern mitochondrial function by controlling shape, subcellular localization, and the mitochondrial network, thereby determining mitochondrial quality. An intricate orchestration of multiple molecular functions ensures the transitions of mitochondrial dynamics. Any deviations from these standard functions can precipitate mitochondrial dysfunction, including the dissipation of mitochondrial membrane potential, malfunction of the ETC, impaired calcium homeostasis, altered oxidative metabolism, and enhanced superoxide production [145]. 

Apart from their vital role in cellular function, mitochondria also serve as an additional source of ROS in cells exposed to CS. CS disrupts the mitochondrial ETC [146], impedes mitochondrial dynamics [147], and undermines the antioxidant system [148], thereby facilitating superoxide generation. A multitude of studies have reported CS-induced mitochondrial ROS formation in cultured cells [146,147,149,150,151]. The most common approach to detect this involves the use of MitoSOX, a mitochondria-targeted fluorescence indicator for superoxide. Additionally, experiments with isolated mitochondria have demonstrated an increase in ROS generation in response to CS exposure [152,153,154]. Notably, mitochondrial ROS levels were found to be elevated in both mice and rats subjected to CS exposure. Furthermore, maternal exposure to CS has been associated with augmented mitochondrial ROS generation in the liver and brain of offspring in both species [155,156]. However, since the expression of manganese SOD (MnSOD), a mitochondrial-specific SOD, was reported to be downregulated in these studies, it remains unclear whether CS-induced oxidative stress is primarily due to enhanced mitochondrial ROS production or a decrease in antioxidant capacity.

The incitement of mitochondrial ROS formation can be primarily attributed to the disruption of the ETC. Evidence suggests that CS alters the molecular regulation of mitochondrial metabolism [146,157]. Studies conducted on isolated mitochondria revealed that CS induced electron leakage and superoxide formation by interfering with electron transfer from complex I to complex III [152,153,154]. Furthermore, CS was found to inhibit the mitochondrial respiratory chain in small airway and bronchial epithelial cells [146]. Moreover, CS impacts the expression levels of ETC components, which adds another layer of complexity. Studies have shown that CS downregulates complexes I, II, III, and IV in small airway epithelial cells [146]. On the contrary, prolonged exposure to CS seemed to upregulate most ETC components in bronchiolar epithelial cells [158]. Alterations in the expression of ETC components, both increases and decreases, have been suggested to result in superoxide production, potentially by enhancing electron leakage, although the causal relationship between these expression levels and superoxide generation remains unclear.

CS has also been found to induce mitochondrial fragmentation-related ROS production in cultured cells. In particular, it was observed that CS increased the expression and activity of a mitochondrial fission protein, dynamin-related protein 1. This augmentation led to the fragmentation of mitochondria and further superoxide formation [147,149,150].

Currently, there is limited information available regarding the molecular mechanisms underlying the elevation of mitochondrial ROS induced by CS. Ca^2+^ seems to play a significant role in this process. The stimulation of Ca^2+^ influx by CS has been linked to mitochondrial fragmentation and MnSOD downregulation, thus promoting mitochondrial ROS production. The primary calcium channels responsible for this Ca^2+^ influx have been identified as TRPA1 and TRPV1 [159]. Additionally, the mitogen-activated protein kinase pathway appears to influence mitochondrial superoxide production induced by CS [160]. For instance, CS was found to trigger mitochondrial superoxide production in bone marrow-derived macrophages through the mediation of mitogen-activated protein kinase kinase 3 (MKK3), an upstream kinase of p38. Mitochondria maintain an efficient antioxidant system to uphold redox balance [151]. However, CS inhibits and downregulates MnSOD, leading to elevated superoxide levels in the mitochondria of cultured cells [148]. MnSOD was also found to be attenuated in the liver and brain of CS-exposed mice and rats [155,156].

MitoSOX, a derivative of dihydroethidium, is commonly employed for the measurement of mitochondrial ROS. It should be noted, however, that the fluorescent signal of MitoSOX arises not solely from superoxide-mediated oxidation but can also be increased by nonspecific oxidation [161,162]. As a result, meticulous studies often employ separation techniques like HPLC to quantify the superoxide-specific product, triphenylphosphonium 2-hydroxyethidium, instead of relying exclusively on the assessment of total fluorescence. Consequently, results obtained using MitoSOX should be interpreted cautiously. For the accurate measurement of mitochondrial superoxide with MitoSOX, appropriate control measures are essential. Notably, CS generates ROS and can induce ROS formation through mechanisms other than provoking mitochondrial dysfunction in cells. Thus, ROS detected in mitochondria might not exclusively originate from this organelle due to the diffusion of ROS. The mitochondrial matrix is not entirely isolated from the cytosolic compartment, enabling ROS to diffuse between the matrix and cytosol [163]. For example, the inner membrane anion channel mediates the efflux of superoxide in mitochondria, though channels or transporters for ROS in mitochondria are not fully deciphered [164]. These considerations are critical in studies involving mitochondria.

#### 3.3.3. NOS

NOS constitutes an enzyme family responsible for catalyzing the conversion of L-arginine to nitric oxide (NO), utilizing O_2_ and NADPH as co-substrates. One notable isoform, endothelial NOS (eNOS), chiefly facilitates the production of NO in the vascular endothelium, a process that demands several cofactors such as heme, Ca^2+^/calmodulin (CaM), flavin adenine dinucleotide (FAD), flavin mononucleotide (FMN), and tetrahydrobiopterin (BH_4_) [165]. During optimal functioning, eNOS promotes electron transfer from NADPH, via FAD and FMN, to the heme iron, assisted by CaM. The now-reduced heme iron subsequently reduces O_2_, a reaction in which BH_4_ serves as a one-electron donor for heme-bound O_2_ [166]. However, under particular conditions, eNOS might divert electrons from NADPH to an O_2_ rather than L-arginine, leading to the generation of superoxide as opposed to NO. This scenario is commonly termed eNOS uncoupling. The uncoupling of eNOS diminishes the NO availability, thereby culminating in endothelial dysfunction [165,167]. This uncoupling can be influenced by various factors, such as S-glutathionylation of eNOS and deficiencies in L-Arg and BH_4_. CS has been particularly implicated in inducing eNOS uncoupling, primarily through the depletion of BH_4_.

Notably, CS incites superoxide formation in endothelial cells with eNOS serving as a source for this superoxide [168]. The generated superoxide, in turn, oxidizes BH_4_ to BH_2_, triggering BH_4_ depletion. This deficiency in BH_4_ instigates eNOS uncoupling, leading to further superoxide production [168,169,170]. As such, a feedforward process is initiated where superoxide derived from CS induces eNOS uncoupling, which consequently generates more superoxide, thereby accelerating eNOS uncoupling [171]. CS-induced eNOS uncoupling can be counteracted by supplementing with SOD and antioxidant vitamins C and E, as well as BH_4_ [168]. 

In addition to regeneration from dihydrobiopterin (BH_2_) via the salvage pathway, BH_4_ is also synthesized de novo from GTP within cells. CS has been found to downregulate GTP cyclohydrolase (GTPCH), a pivotal rate-limiting enzyme involved in BH_4_ biosynthesis, thereby inhibiting BH_4_ synthesis. This downregulation is attributed, at least in part, to accelerated degradation via the ubiquitin-proteasome system [172].

The evidence of CS-induced eNOS uncoupling has been gathered from both in vivo animal studies and clinical studies involving smokers [169,173,174,175]. In mouse models, CS induced endothelial dysfunction by depleting BH_4_ and provoking eNOS uncoupling. The upregulation of gp91phox and p22phox and the subsequent elevation in superoxide by CS have been implicated as potential drivers of oxidative stress, leading to BH_4_ depletion [176]. The administration of BH_4_ ameliorated endothelial dysfunction in chronic smokers [169], despite no significant difference being noted in basal plasma levels of BH_4_ between smokers and nonsmokers [177].

Information remains scarce regarding CS-induced uncoupling of neuronal NOS (nNOS) or inducible NOS (iNOS), which are other isoenzymes of NOS. Instead, CS can induce the expression of iNOS in inflammation, resulting in the production of nitric oxide and reactive nitrogen species [178]. Regardless of uncoupling, CS exhibits an inhibitory effect on NOS. Specifically, CS has been shown to attenuate eNOS activity by shifting eNOS phosphorylation to an inhibitory state in a PKC-dependent manner [170]. Moreover, CS directly inhibits nNOS by interacting at the substrate binding site [168,179]. The pathophysiological significance of these observations is currently uncertain. CS has been found to upregulate iNOS through a ROS-dependent deactivation of the Nrf2-SIRT3 signaling axis in mice and cells [180]. This CS-induced iNOS, along with ROS, has been implicated in the ferroptosis of the bronchial epithelium.

#### 3.3.4. NF-κB and Sirtuin 1 (SIRT1), as Regulators for ROS Sources

Apart from NOX, mitochondria, and NOS, cells harbor other endogenous sources of ROS. These include but are not limited to xanthine oxidase, cyclooxygenase, lipoxygenase, and CYP [181]. Despite their established role in ROS production, the degree to which these sources contribute to CS-induced ROS formation remains less well explored. The limited evidence available thus far precludes a definitive conclusion as to whether CS indeed does not engage these sources in ROS production or if they have merely been under-investigated. 

The regulatory dynamics between the SIRT1 and NF-κB systems provide a conduit for potential indirect sources of ROS. Through their mutually antagonistic interactions, these systems orchestrate metabolic and inflammatory responses [182]. Despite not being direct ROS sources, their downstream signals can instigate ROS production from established cellular sources, including NOX and mitochondria.

CS is known to attenuate SIRT1 activity through a reduction in its expression levels [183,184,185,186,187]. In contrast, the activity and expression of NF-κB are augmented by CS. Under normal conditions, SIRT1 mitigates ROS production from mitochondria and NOX and further prevents eNOS uncoupling by upregulating GTPCH1 [188]. Hence, the CS-induced downregulation of SIRT1 potentiates cellular ROS production. This downregulation of SIRT1 has been attributed to posttranslational modifications, triggering accelerated proteasomal degradation [188]. NF-κB, on the other hand, modulates the transcriptional regulation of several ROS sources including NOX, xanthine oxidase, lipoxygenase, and cyclooxygenase-2 [181,189]. Therefore, its activation by CS indirectly promotes ROS generation within cells. In lung fibroblasts, CS was shown to augment mitochondrial ROS by inducing NOX4 expression and inhibiting MnSOD activity within mitochondria. The hsa_circ_0006872-miR-145-5p axis has been suggested as a potential mediator of CS-induced NF-κB activation, although the precise molecular mechanisms remain to be fully elucidated [190]. Observations in vivo corroborate these findings. For instance, the inhibition of SIRT1, the activation of NF-κB, and the subsequent rise in ROS were observed in arteries and hearts of CS-exposed rats [183]. Similarly, decreased SIRT1 expression and increased RelA/p65 NF-κB expression, coupled with elevated levels of 4-HNE and 3-nitrotyrosine, were detected in lung tissues from smokers [186]. Taken together, these findings suggest that the SIRT1-NF-κB signaling axis plays a pivotal role in mediating the effect of CS on cellular ROS production.

### 3.4. Impairment of Antioxidant System by CS

An essential determinant of the cellular redox state is the intricate antioxidant system, which serves to counterbalance or mitigate the presence of prooxidants. The antioxidant system comprises both enzymatic and non-enzymatic elements [5]. Enzymatic components include SOD, catalase, glutathione peroxidase, thioredoxin, and peroxiredoxin, whereas non-enzymatic counterparts comprise vitamin C, vitamin E, carotenoids, and GSH. Even though the antioxidant system does not generate ROS directly, its status profoundly influences ROS concentrations. Consequently, when discussing the impact of CS on ROS, the corresponding effect on the antioxidant system must be considered. 

A multitude of prooxidants present in CS exert varied effects on antioxidant capacity, thereby disrupting the integrity of the antioxidant system [55,191]. Independent of ROS production, CS compromises the antioxidant system by suppressing or downregulating enzymatic antioxidants and depleting non-enzymatic antioxidants. However, in the majority of observations, antioxidant levels remain stable or are even elevated in subjects exposed to CS [53,192]. Such a phenomenon is typically interpreted as an adaptive response to oxidative stress. Initially, oxidative stress typically induces a decline in antioxidant capacity, which is subsequently followed by an upregulation of antioxidants in a compensatory or rebound response to oxidative stress [193]. Despite this, the primary response to smoking or CS exposure undeniably involves a decrease in antioxidant capacity. 

Analyses have revealed that the activity and expression levels of SOD and catalase are diminished in alveolar macrophages [191], erythrocytes [55], circulating progenitor cells [194], and blood serum [56] of smokers. Consistent findings were observed in murine models and cellular studies. The downregulation of SOD was shown to be a consequence of SIRT1 inhibition or repression by CS [184,185,187]. In cell-free systems, CS was demonstrated to directly inhibit SOD and catalase [195]. 

CS has been shown to decrease the total GSH in cells by forming GSH adducts [196,197]. Reactive aldehydes such as acrolein, α,β-unsaturated ketones, and methyl vinyl ketones, all constituents of CS, undergo GSH conjugation. This reaction irreversibly forms conjugates with GSH, thus leading to GSH consumption [198]. CS also impacts GSH metabolism by downregulating glutamate cysteine ligase, a rate-limiting enzyme in GSH synthesis, which results in a decrease in GSH [199,200,201]. Furthermore, CS inhibits and downregulates GSH-S-transferase and GSH peroxidase, enzymes which catalyze the conjugation of GSH to xenobiotic substrates and the reduction in hydrogen peroxide or peroxide radicals, respectively [55,191,194,199]. Thus, CS depresses GSH levels by amplifying its consumption and repressing its synthesis or regeneration.

## 4. Additional Issues and Perspectives

Mounting evidence suggests that CS may induce the formation of ROS within biological systems. Nevertheless, several salient issues warrant further consideration to enrich our understanding of the relationship between CS and ROS.

### 4.1. Clinical Relevance of In Vitro Findings

Most research on the induction of ROS formation by CS has been predominantly conducted using in vitro systems, a common approach in mechanistic studies. As such, it remains uncertain as to whether these observations reliably translate to in vivo contexts or human subjects. The sheer complexity of CS content, encompassing several thousand chemicals with diverse bioavailability, complicates the task of pinpointing the specific constituents responsible for ROS formation [18,202]. Thus, it remains an open question whether the constituents that trigger ROS formation in in vitro systems retain their effectiveness in more complex biological environments.

A further pivotal factor to consider is the degree of exposure. Ideally, treatments with CS preparations should accurately emulate conditions experienced during smoking. The biological activities of chemicals are inherently contingent on their concentration or dosage. For instance, depending on its concentration, CS can either inhibit or stimulate cell proliferation [203], a dynamic likely mirrored in cytokine production, which can be either suppressed or activated [204]. Moreover, the composition of CS preparations does not precisely match that of original CS. Although the air–liquid interface in vitro models may partially circumvent this issue, these models also present technical challenges for real-time ROS measurements. Currently, no consensus exists regarding the optimal concentration of CS preparation for experimental treatment. Some studies have attempted to equate marker compounds, such as nicotine, to concentrations observed in smokers. However, this approach lacks precision due to the variable bioavailability of CS constituents, including these marker compounds [202]. Using concentrations that are unrealistically high may yield invalid results. Therefore, experimental outcomes must be interpreted in light of their potential clinical relevance. To aid in the evaluation and comparison of results, the standardization of concentration expression—for example, the number of cigarettes, μg/mL TPM, puff/L, or percentage of CSE—is imperative at the outset of any study.

### 4.2. Bioavailibility of ROS and Radicals in CS

As discussed previously, the majority of ROS and radicals exhibit a high degree of reactivity and short half-lives [80]. Remarkably, the respiratory system is equipped with a sophisticated antioxidant system [205]. For example, the human epithelial lining fluid is characterized by extraordinarily high levels of GSH [206], and alveolar macrophages exhibit a robust GSH-dependent peroxide metabolism system [207,208]. Consequently, not all ROS or radicals originating from CS are available systemically. This fact contributes to the reason why not all in vitro observations are likely to translate directly into in vivo contexts. At present, information regarding the toxicokinetics of exogenous ROS or radicals is insufficient, thus significantly limiting our understanding of these processes.

### 4.3. ROS Detection Methods Compatible with CS

The study of the relationship between CS and ROS necessitates robust methods for ROS detection. However, the accurate quantification of ROS remains a significant challenge due to the inherent chemical properties of ROS, including their high reactivity and short lifespan. The limitations of ROS indicators, such as their limited specificity and the complexity of the associated chemical reactions, can further complicate the process [209]. While ESR combined with spin trap is generally regarded as the most reliable method for detecting a range of ROS with specificity, its application in biological systems is not without technical limitations [210]. In practice, optical probing methods, using colorimetric, fluorescent, or luminescent indicators, are frequently employed to detect ROS. Despite their utility, these methods bear their own limitations and face additional challenges when applied to CS. Notably, CS preparations exhibit inherent autofluorescence and optical absorbance across a broad wavelength spectrum, rendering them incompatible with various fluorophores [116]. For instance, CSE and TPM derived from standard reference cigarettes have been shown to interfere with optical signals from chemiluminescent probes, such as luminol, and fluorescent indicators, such as 2′,7′-dichlorodihydrofluorescein and Amplex red, due to quenching or autofluorescence [116]. Moreover, CS has the potential to inhibit horseradish peroxidase, a common element used in conjunction with probes like luminol and Amplex red for oxidation reactions [116,211]. These experimental artifacts hinge on the composition of the CS preparations, which is influenced by the nature of the CS products and the methods used in their preparation. The concentration of CS preparations tested can also impact results. As such, it is recommended to preliminarily examine the optical properties of CS preparations to circumvent these obstacles. ROS detection in biological systems poses a more complex challenge than in in vitro systems. Despite the fact that ROS has been measured in tissue homogenates or cryosections, the interpretation of these results should be undertaken with caution, given the short half-lives of ROS and potential artifacts induced by tissue manipulation such as homogenization or freezing, which could influence substrate or redox metal ions [6]. In order to validate experimental results, orthogonal approaches, as suggested in the literature, are recommended [6].

### 4.4. Emerging Alternative Smoking Products: Heated Tobacco Products (HTPs) and Electronic Cigarettes (E-Cigarettes)

HTPs, also known as heat-not-burn products, are the electronic tobacco-containing devices that heat tobacco instead of burning it, producing inhalable aerosols that contain nicotine and other chemicals. E-cigarettes, an electronic nicotine delivery system, heat an “e-liquid” that typically contains nicotine, as well as flavorings such as propylene glycol and glycerin, and other ingredients, to generate aerosols. These products gained popularity based on the perception that they pose a reduced risk compared to traditional cigarettes.

The emissions from HTPs are generated by heating tobacco at lower temperature than combustion, containing a reduced amount of tar [212]. The constituents of aerosols from E-cigarettes are notably distinct from those of emissions from tobacco products due to the use of e-liquid instead of tobacco leaves [213]. Nonetheless, these still exhibit similar characteristics that impose ROS burden. Similar to conventional cigarettes, emissions from HTPs and E-cigarettes induce oxidative stress, as evidenced by the detection of oxidative stress markers in experimental animals [214,215,216]. ROS were detected in the emissions from them [216,217]. These emissions have the potential to incite ROS production in cells, with NOX and mitochondria acting as cellular sources for ROS [218,219,220,221,222]. However, there appears to be a distinction in the quantitative aspect of their capacity to induce ROS formation. 

In the comparative studies, emissions from HTPs were found to contain a lower quantity of ROS compared to CS [79], and both HTPs and E-cigarettes demonstrated a lower ability to stimulate cells to produce ROS compared to conventional cigarettes [220,223,224,225,226]. Nevertheless, E-cigarettes induced a comparable alteration in oxidative stress makers to CS in mice studies [227]. Presently, it remains uncertain whether alternative smoking products have a lower potential to induce ROS formation and share similar molecular mechanisms for ROS production with conventional cigarettes. Given the limited information and insufficient clinical studies available, this review did not cover alternative smoking products. Nonetheless, the growing popularity of these products underscores the need for ongoing attention.

### 4.5. Beyond the Current Review

With a focus on the topic of how CS imposes a burden of ROS on biological systems, this study primarily reviewed the processes for ROS formation, rather than delving into the subsequent biological consequences. This is not due to the insignificance of the biological activities of ROS, but rather because an extensive body of literature on this topic is already available elsewhere. In addition to ROS, there are other types of reactive species that perturb redox balance, which encompass reactive nitrogen species, reactive carbonyl species, reactive sulfur species, and non-ROS radicals [228,229,230,231]. Covering all these entities within a review would be too voluminous work. Despite the existence of other tobacco products like smokeless tobaccos, HTPs, and E-cigarettes, the information reviewed here is confined to studies involving conventional cigarettes, because conventional cigarettes are the most popular and extensively studied form of tobacco products. Exploring topics beyond the scope of this review would hold value for forthcoming studies.

## 5. Conclusions

ROS serve as common pathogenic mediators in the development of health problems caused by smoking. There are ROS inherently present in CS. Additionally, CS induces ROS formation through chemical reactions between its constituents and biomolecules, and stimulates cellular ROS sources to produce ROS. Furthermore, CS disrupts the antioxidant systems, influencing the redox state and consequently amplifying ROS functions. Collectively, all these ROS contribute to the ROS burden placed by CS exposure (Figure 1).

As delineated in the introductory section, it is of paramount importance to elucidate the mechanisms by which CS incites the formation of ROS. This understanding not only enhances our comprehension of the molecular events precipitating pathogenesis but also facilitates the establishment of strategic interventions designed to mitigate the oxidative stress induced by smoking. Efforts to augment this existing knowledge base will undoubtedly be highly valued in the associated scientific disciplines.

## Data Availability

No new data were created or analyzed in this study. Data sharing is not applicable to this article.
